# A New Simulated Plasma for Assessing the Solubility of Mineral Trioxide Aggregate

**Published:** 2014-12-24

**Authors:** Mohammad Samiei, Shahriar Shahi, Naser Aslaminabadi, Hadi Valizadeh, Zahra Aghazadeh, Seyyed Mahdi Vahid Pakdel

**Affiliations:** a * Department of Endodontics, Tabriz University of Medical Sciences, Tabriz, Iran; *; b * Department of Pediatric Dentistry, Tabriz University of Medical Sciences, Tabriz, Iran; *; c * Department of Pharmaceutics, Tabriz University of Medical Sciences, Tabriz, Iran;*; d * Department of Oral Medicine, Tabriz University of Medical Sciences;*; e * Student Research Committee, Department of Prosthodontics, Tabriz University of Medical Sciences*

**Keywords:** Blood Substitute, Mineral Trioxide Aggregate, MTA, Plasma, Solubility

## Abstract

**Introduction:** Solubility of mineral trioxide aggregate (MTA) is an important characteristic that affects other properties such as microleakage and biocompatibility. Distilled water (DW) has previously been used for solubility tests. This experimental study compared the solubility of MTA in DW, synthetic tissue fluid (STF) and new simulated plasma (SP). **Methods and Materials:** In this study, 36 samples of tooth-colored ProRoot MTA were prepared and divided into three groups (*n*=12) to be immersed in three different solutions (DW, STF, and SP). Solubility tests were conducted at 2, 5, 9, 14, 21, 30, 50, and 78-day intervals. The unequal variance F-test (Welch test) was utilized to determine the effect of solubility media and Games-Howell analysis was used for pairwise comparisons. The repeated-measures ANOVA was used to assess the importance of immersion duration. **Results: **Welch test showed significant differences in solubility rates of samples between all the different solubility media at all the study intervals (*P*<0.05) except for the 14-day interval (*P*=0.094). The mixed repeated-measures ANOVA revealed a significant difference in solubility rate of MTA in three different solutions at all time-intervals (*P*=0.000). Games-Howell post-hoc test revealed that all pairwise comparisons were statistically significant at all time-intervals (*P*=0.000). **Conclusion: **Based on the findings of this study, the long-term solubility of MTA in simulated plasma was less than that in synthetic tissue fluid and distilled water.

## Introduction

Solubility of materials used in endodontic therapy affects the treatment prognosis [1]. Solubility rate of a dental material is one of the properties that can probably affect other features like sealing ability and biocompatibility [2, 3]. According to International Association of Standardization (ISO), the solubility rate should be under 3% of the whole mass [4]. If a root-end filling material disintegrates progressively in periradicular tissue fluids, subsequent leakage would lead to treatment failure [5, 6].

Due to low solubility, low cytotoxicity, tissue biocompatibility, and ability to induce mineralized tissue formation, mineral trioxide aggregate (MTA) is indicated to seal perforations and as a retrograde filling material, as a pulp capping agent, for repair of external and internal root resorptions, apexification and root canal sealer [7]. MTA is a bioactive material which induces hard tissue formation. It has low solubility, low cytotoxicity, tissue biocompatibility, and ability to induce mineralized tissue formation. One of the byproducts of MTA and water is calcium hydroxide (CH) which is soluble in distilled water (DW) and is a major factor in MTA solubility [8]. In contact with tissue fluids, CH is converted to hydroxyapatite (which is water-insoluble) and deposits on MTA surface. Reduction in the microleakage of MTA with the pass of time has been attributed to this phenomenon [3].

In most previous studies on the solubility of MTA, ISO and American Dental Association (ADA) approaches have been employed, in which the solubility of root canal sealing materials has been assessed in DW [4, 8-12]. Saghiri *et al.* [13], assessed the solubility of MTA in deionized water and synthetic tissue fluid (STF). However, none of above-mentioned studies mimicked compositional condition of blood adjacent to the filling material. In clinical practice and as a root-end filling material, MTA comes in contact with tissue fluid and blood. As tissue fluid and human blood better mimic the dental surrounding media, it seems that they would act as better solutions to test the solubility of a root-end filling material which comes in contact with them during and after surgery. Kintner *et al.* [14] used blood substitute [simulated plasma (SP)] for maintaining isolated canine brain. It was the first time that blood substitute has been used in medical conditions instead of blood. In dental field there is not such a research to use blood substitute. Handling human blood needs an anticoagulant agent (*e.g.* heparin) and some other considerations that may interfere with the study results. Therefore in the present experimental study, the solubility of MTA was assessed in STF, SP as well as DW.

## Materials and Methods

The solubility of tooth-colored ProRoot MTA (Dentsply, Tulsa Dental, Tulsa, OK, USA) in three different solutions was assessed according to the ISO 6876 standard [4] and the ADA specification [15] with minor modifications. The solubility was periodically determined at increasingly longer periods of time, *i.e.* at 2, 5, 9, 14, 21, 30, 50, and 78-day intervals.

According to the ISO standard [4], 36 metal molds (20 mm in diameter and 2 mm in height) were numbered using a diamond bur on the mold circumference and weighed with analytic scale (AND GR-200 Analytical Balance, Lab Recyclers Inc., Gaithersburg MD, USA) with 0.1-mg precision. The samples were mounted on glass slabs with sticky wax. Twelve sets of specimens were prepared for each solution in one step by one operator. To this end, MTA was mixed with DW according to the manufacturer’s recommendations and packed into the mounted rings and the surfaces were flattened by means of a glass slab. The samples were handled at room temperature (23±2^°^C) and 60±5% relative humidity. For moist curing, the samples were then placed in a heating chamber for 21 h at 37±1^°^C and 100% relative humidity for initial setting. Once removed from the chamber, the specimens were dried in a desiccator attached to a vacuum machine until they reached a constant weight; this step is necessary to eliminate all the free water from the samples to achieve a consistent net weight reference. Each sample was weighed twice. To minimize room humidity absorption, this process was accomplished in less than 20 sec. After subtraction of rings’ weight from the latter weight, the resultant weight was recorded as initial dry weight.

**Table 1:** Composition of synthetic tissue fluid

**Ingredient**	**mg/100 mL**
**Potassium hydrogen phosphate**	17
**Disodium hydrogen phosphate**	118
**Sodium chloride**	800
**Potassium chloride**	20

The samples were randomly divided into three groups to be immersed in three different solutions (*i.e.* DW [pH=7.2], STF, and SP). The compositions of STF and SP are shown in Tables 1 and 2, respectively.

Solubility tests were conducted inside an incubator at 37^°^C. At the predetermined time intervals of 2, 5, 9, 14, 21, 30, 50, and 78 days the solubility media containing dissolved samples were collected and the specimens were immersed in other bottles with 50 mL of corresponding fresh solubility medium (37^°^C). The collected solubility media were then evaporated at 90‒95^°^C. Bottles and residues were re-dried in an oven at 105‒110^°^C, cooled down in a desiccator and re-weighed. In DW samples, differences between the final weight and the original bottle weight were expressed as the specimens’ initial dry weight percentages. According to the ISO 6876 standard, these values are called “solubility”. In STF and SP samples, the final dry weight was subtracted from STF/SP dry ingredients and then the initial dry weight was calculated.

**Table 2:** Composition of simulated plasma according to Kintner *et al. *   [14]

**Ingredient**	**Amount (g/1000 mL)**
**Isoleucine **	5.10
**Leucine**	8.90
**Lysine**	5.60
**Methionine**	3.80
**Phenylalanine**	5.10
**Threonine**	4.10
**Tryptophan**	1.80
**Valine**	4.80
**Arginine**	9.20
**Histidine **	5.20
**Glycine**	7.90
**Alanine**	13.70
**Proline**	8.90
**Aspartic acid**	1.30
**Asparagine monohydrate**	3.72
**Cystine**	0.50
**Glutamic acid**	4.60
**Ornithine**	2.51
**Serine**	2.40
**Tyrosine**	0.30
**Acetyl tyrosine**	1.23
**Albumin**	40
**Sodium acetate**	3.95
**Potassium acetate**	2.45
**Magnesium acetate**	0.56
**Sodium hydrogen phosphate**	1.40
**Sodium hydroxide**	0.20
**Malic acid**	1.01

The Levene analysis was used for homogeneity of variances at different time intervals. The Unequal variance F-test (Welch test) and repeated-measures one-way ANOVA were used to compare the solubility in different media and different time intervals, respectively. The Games-Howell post-hoc test was used for pairwise comparisons. The SPSS software (SPSS version 17.0, SPSS, Chicago, IL, USA) was used for all the statistical analyses. The level of significance was set at 0.05.

## Results

Statistical data are presented in Table 3. The Kolmogorov-Smirnov analysis revealed normal distribution of solubility rate of tested materials in all the three solutions at all study intervals (*P*>0.05). The Levene statistics for testing the homogeneity of variances depicted unequal variances at all time-intervals (*P*<0.05). The Unequal variance F-test (Welch test) showed a significant difference in solubility rate in tested solubility media at all time-intervals (*P*<0.05) except for the 14-day interval (*P*=0.094). The Games-Howell post-hoc test was used for pairwise comparisons. Results of pairwise comparisons in definite groups are presented in Table 3.

**Figure 1:**
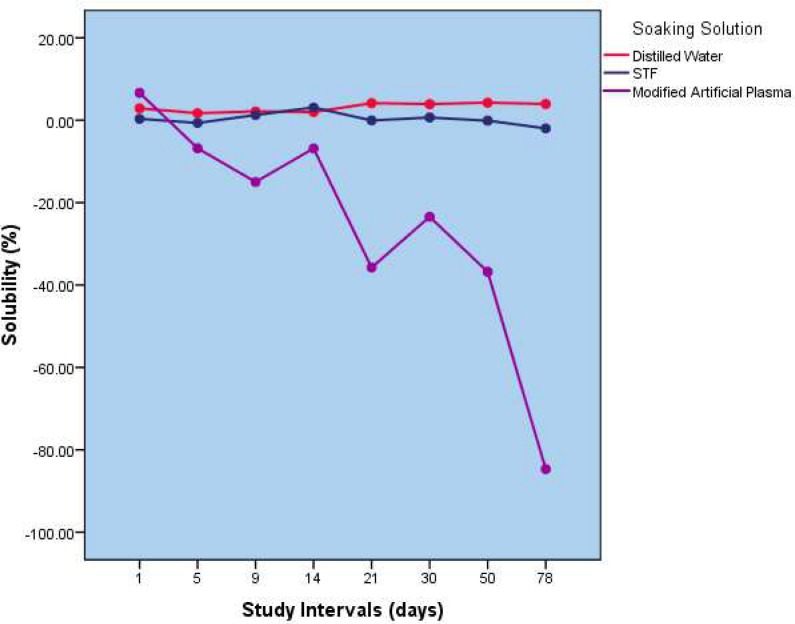
Comparison of solubility (%) of MTA in three different solubility media as a function of time

The repeated-measures ANOVA revealed a significant difference in solubility rate of MTA in three different solutions at all the study intervals (*P*=0.000). The Games-Howell post-hoc test revealed that all pairwise comparisons were statistically significant at all time-intervals (*P*=0.000) (Figure 1).

## Discussion

This experimental study evaluated the solubility of MTA in DW, STF and SP in different time-intervals and showed that the long-term solubility of MTA in SP is less than that of STF and DW.

The aim of employing a root-end filling material is to prevent microleakage of irritants from the root canal system to periradicular tissues. Therefore, an ideal root-end filling material should provide apical seal and be non-toxic, tolerated by periradicular tissues, insoluble and dimensionally stable, easily manipulated, radiopaque and have some bactericidal or bacteriostatic activity [16].

Solubility of a root-end filling material is one of its utmost important features since it would influence other properties like sealing ability, biocompatibility, and mutual effects with surrounding environment [2, 3]. Sealing ability and solubility could affect treatment prognosis [1]. The root-end filling material should have very low solubility because the materials released from it might have toxic effects on surrounding tissues [2]. According to ISO, the dissolved amount should be less than 3% of the whole mass [4].

MTA is a mixture of dicalcium silicate, tricalcium silicate, tricalcium aluminate, tetracalcium aluminoferrite, and bismuth oxide. It contains other elements such as SiO_2_, CaO, MgO, Al_2_O_3_, K_2_SO_4_, FeO, and Na_2_SO_4_. Due to low solubility, low cytotoxicity, tissue biocompatibility, and the ability to induce mineralized tissue formation, MTA has been used in vital pulp therapy (direct pulp capping, partial pulpotomy, and pulpotomy), repair of furcal and lateral perforations, as an apical plug in immature non-vital teeth and as a root-end filling material during apical surgery [17-20].

Different studies have addressed the solubility of MTA and compared it with ISO method [8, 9, 11, 12, 21, 22]. In ISO 6876 and ADA protocol, the solubility of dental materials are measured in DW and the solubility of ≤3% is acceptable [4, 15, 23]. In the majority of previous studies the solubility of materials has been measured only in DW (according to ISO method), the solution which does not have any similarity with the composition of periradicular tissues [8-12]. However, MTA is a biomaterial and has interactions with surrounding vital environment when in contact with biologic tissues and fluids. Therefore, solubility tests of these materials should be performed in physiologic solutions with similarity to periradicular tissue fluids. As tissue fluid and human blood better mimic the dental surrounding media, it seems that they would act as better solutions to test the solubility of a root-end filling material which comes in contact with them during and after surgery. In a study conducted by Saghiri *et al. *[13], solubility of MTA was investigated in deionized water and STF. They reported less solubility rates of MTA in STF than deionized water and suggested STF as an alternative to distilled water for solubility studies of dental materials due to similarity of STF to biological fluids. 

**Table 3:** Mean (SD) of solubility of tested materials in three different solutions at different time-intervals.

**Storage medium**	**Solubility mean (SD)**							
	**Day 1**	**Day 5**	**Day 9**	**Day 14**	**Day 21**	**Day 30**	**Day 50**	**Day 78**
**Distilled water**	2.8984^a^ (0.5090)	1.7117 (0.9981)	2.1391^a^ (0.4361)	2.0093^a,b^ (1.0688)	4.1494^a,b^ (0.8338)	3.9094^a^ (0.6126)	4.2544^a^ (0.7035)	3.9446^a,b^ (1.0956)
**Synthetic tissue fluid**	0.3338^a,b^ (1.6547)	-0.6520 (4.3065)	1.2695 (0.4876)	3.0872^a, c^ (1.8304)	-0.0474^a,c^ (1.5847)	0.6693^a^ (1.3077)	-0.0993^b^ (3.1806)	-1.9859^a,c^ (3.4446)
**Simulated plasma**	6.6518^b^ (5.3727)	-6.7991 (8.4592)	-14.9807^a^ (9.2213)	-6.8456^b,c^ (19.9333)	-35.8647^b,c^ (19.1879)	-23.4304 (42.6082)	-36.7835^a,b^ (31.3863)	-84.6802^b,c^ (21.9376)

*Groups with the similar letter are significantly different in pairwise comparison at each time interval; the minus mark illustrates the formation of some deposits on the material surface*

The method of measuring dissolution rate in most reported studies involves determination of the weight difference of the biomaterial before and after dissolution [8, 11, 12, 24]. Although this method is presented as a standard procedure, in fact it is indicative of the wash-out of the material in solution rather than actual solubility. Solubility of a material is defined as the amount of material which dissolves in a certain amount of a solvent. Therefore, the measurement of weight difference of the material before and after dissolution might not show the actual solubility since it is possible that some cement disintegrates without any dissolution [25, 26]. In other words, the result obtained with this method is the combination of solubility and disintegration rates. Due to the similar effect of these two phenomena (solubility and disintegration) on the properties of root-end filling materials (sealing ability, biocompatibility, *etc.*), there is no need for measuring these exclusively and the measurement of the combination rate is also adequate and critical.

According to ISO protocol, solubility test should be carried out 24 h after material setting. In the present study, 2 to 78-day intervals were used to evaluate the effect of longer time periods on the dissolution process. Some previous studies have employed similar methods, evaluating the dissolution rate at 28- and 78-day intervals as well as ISO protocol [9-12].

In this study, desiccant materials in a vacuum-attached desiccator (for elimination of environmental humidity) were utilized for drying specimens before dissolution to achieve a constant weight in each one. In some previous studies, the samples were put in a temperature of 105^º^C for this purpose [8]. Since the latter would alter the material’s crystalline structure and interfere with measuring dissolution rate, it was not employed in the present study. In a pilot study, the latter method had destructive effects on the samples.

Fridland and Rosado [8], reported that the higher is the water-to-powder ratio of MTA, the higher is the solubility rate. Therefore, we used the powder-to-water ratio recommended by the manufacturer to eliminate the effect of such disruptive factors.

It has been reported that the solubility of MTA decreases with time but never reaches zero [9, 11]. In this study, the solubility rate had a descending pattern but it was somehow irregular. Since the solubility and disintegration phenomena take place inevitably and none of the pioneer studies have measured these exclusively, and because the disintegration is an unpredictable process, its irregular mode seems natural.

It seems that STF and SP (particularly SP) are more similar to periradicular tissue fluids and solubility studies on these media show the actual solubility of material in comparison to distilled water. Therefore, it would be better to evaluate the solubility of biomaterials in these solutions.

## Conclusion

Based on the findings of this experimental study, the solubility of MTA in modified artificial plasma was less than that in synthetic tissue fluid and distilled water in the long-term.
